# Preface to the special topic on genome editing research in China

**DOI:** 10.1093/nsr/nwz054

**Published:** 2019-06-14

**Authors:** Jinsong Li, Caixia Gao

**Affiliations:** 1 CAS Center for Excellence in Molecular Cell Science, Shanghai Institute of Biochemistry and Cell Biology, Chinese Academy of Sciences, China; 2 Center for Genome Editing, Institute of Genetics and Developmental Biology, Innovation Academy for Seed Design, Chinese Academy of Sciences, China ^3^University of Chinese Academy of Sciences, China

Genome engineering technologies enable the precise modification, regulation and tagging of genomic loci in living cells and organisms, leading to a broad range of applications from basic biology to biotechnology and biomedicine. For many years, precise genome manipulation has heavily relied on the homologous recombination (HR)-based gene targeting strategy, which has been limited to several organisms (yeast, mouse and human). However, recent revolutionary findings on bacterial RNA-guided clustered regularly interspaced short palindromic repeat (CRISPR)-Cas systems have led to a range of simple, fast and efficient genome engineering technologies, which are applicable to a wide range of organisms, including species that are recalcitrant to genetic manipulation via the traditional HR-based method. With its rapid development over the past few years, CRISPR-Cas9 and its variants have become the most powerful and versatile platforms for genetic and epigenetic editing, and can efficiently fulfill targeted genome editing, gene expression regulation, genome imaging, epigenetic modifications, base mutations and functional genomic screening. Obviously, CRISPR-Cas9 technologies, after overcoming the difficulties in genomic modifications that used to cause a bottleneck in the post-genomic era, will pave the way for the progress of functional genomics and promote its broad application in biotechnology and biomedicine, such as in the generation of disease models to reveal pathological mechanisms and enable drug discovery, the enhancement of crop and livestock production to obtain high-quality agricultural products, the construction of transgenic bioreactors to obtain bioproducts at low cost such as biofuels and the development of genetic therapies to cure human diseases.

Since the advent of CRISPR-Cas9 in 2013, genome editing has become one of the most rapidly growing areas in science and has drawn much attention in recent years. Chinese scientists have played a significant role in promoting the development of the field by making enormous achievements over the last 5 years, such as the first generation of targeted gene-modified model organisms, which had previously been extremely difficult to achieve, including rat, zebrafish and monkey, and the development of human disease models using large animals such as non-human primates and pigs, the correction of genetic defects in mice, the establishment of CRIPSR-Cas9-mediated high-throughput screening systems for functional genomics, successful genome editing in insects, efficient genome modification of crop plants, revealing the structures of CRISPR-Cas systems and so on.

To reflect the state-of-the-art nature of genome editing research in China, this special topic of *National Science Review* presents three timely review articles and three insightful perspectives, along with three interesting research highlights and a valuable interview. The review article by Yanfei Mao and colleagues discusses the progress and challenges in genome editing in plants, which has a long history with remarkable recent progress. The review article by Jianguo Zhao and co-authors describes an overview of the current state and future prospects of genome editing in large animals, which is critical for human disease modeling, regenerative medicine and the improvement of agricultural livestocks. The review article by Yuwei Zhu and Zhiwei Huang focuses on the recent advances in structural studies of CRISPR-Cas, which is regarded as an important basis for the efficient application of CRISPR-Cas as a genome editing tool. In their perspective article, Jun Xu *et al*. discuss the current state of research and challenges regarding genome editing in insects, which has been greatly enhanced by the development of CRISPR-Cas9 technologies. The perspective article by Zhuo Zhou and Wensheng Wei focuses on high-throughput CRISPR-Cas9-mediated genetic screening for non-coding genomes, which is a powerful approach for interrogating the biological and physiological functions of non-coding genetic elements. The perspective article by Jiang Jing and co-authors proposes a huge project, referred to as genome tagging project, based on ‘artificial spermatids’ and CRISPR-Cas9 technologies, in which all protein coding genes will be endogenously tagged in mice using a standard tag. Three research highlights introduce recent key advances achieved by Chinese scientists in genome editing research. The research highlight by Feng Zhang and Daniel F. Voytas reports a recent significant advance in genome editing in plants, in which gene expression has been successfully regulated at the translational level through the editing of upstream open reading frames. Chengzu Long highlights recent achievements in precise and predictable CRISPR-Cas9-mediated DNA fragment editing, which may lead to a higher chance of obtaining desired editing outcomes. The highlight article by Yuyu Niu discusses the *SIRT6* mutant monkey generated by zygote injection of CRISPR-Cas9, representing a key advance in genome editing in large animals. Finally, an interview with David Liu, who is the inventor of the CRISPR-Cas-mediated base editor technology, is also included in this special topic to share his personal views on CRISPR-Cas technology and prospects for the field of genome editing science.

As the guest editors, we would like to express our sincere appreciation to all of the authors, reviewers and also the editorial staff at *National Science Review* for their time and dedication, which has made this special topic possible. We hope that this special topic will receive broad attention from researchers working in multiple disciplines including, but not limited to, genome editing, basic biology and plant, animal and medical sciences.

